# Demenzen: Änderungen von ICD‑10 zu ICD-11

**DOI:** 10.1007/s00115-025-01884-w

**Published:** 2025-09-24

**Authors:** Frank Jessen, Karl Broich

**Affiliations:** 1 Klinik und Poliklinik für Psychiatrie und Psychotherapie, Medizinische Fakultät, Uniklinik Köln, Kerpener Straße 62, 50937 Köln, Deutschland; 2 Deutsches Zentrum für neurodegenerative Erkrankungen (DZNE), Köln/Bonn, Deutschland; 3 Bundesinstitut für Arzneimittel und Medizinprodukte (BfArM), Bonn, Deutschland

**Keywords:** Schweregrade, Postkoordination, Alzheimer-Krankheit, Leichte neurokognitive Störung, Biomarker, Severity level, Postcoordination, Alzheimer’s disease, Mild neurocognitive disorder, Biomarkers

## Abstract

Die International Statistical Classification of Diseases and Related Health Problems 11 (ICD-11) stellt einen konzeptuellen Fortschritt zur ICD-10 bei der Klassifikation von Demenzen dar. Die syndromale Einteilung im Kapitel Neurokognitive Störungen bleibt zwar prinzipiell erhalten, die Einführung von Schweregraden und die zentrale Codierung von psychischen und Verhaltensstörungen erlauben aber eine präzisere Erfassung der klinischen Diagnosen. Neu ist ferner die leichte neurokognitive Störung als Frühstadium von Demenzerkrankungen. Noch nicht ausreichend aufgenommen wurden die von internationalen Experten erarbeiteten klinischen Kriterien wie z. B. für die frontotemporale Demenz oder die Lewy-Körper-Krankheit. Auch Biomarker für die ätiologische Diagnostik von Demenzen werden nicht aufgeführt, sodass unklar bleibt, welche Rolle sie bei der Krankheitszuordnung in ICD-11 spielen. Aufgrund der schnellen Entwicklung im Feld neurodegenerativer Erkrankungen wären regelmäßige Aktualisierungen wünschenswert.

## Abstract (en)

The International Statistical Classification of Diseases and Related Health Problems version 11 (ICD-11) represents a conceptual advance over ICD-10 in the classification of dementias. Although the syndromic classification in the chapter “Neurocognitive disorders” remains in principle unchanged, the introduction of severity levels and the central positioning of mental and behavioral symptoms enables a more precise coding of the clinical diagnoses. Furthermore, the introduction of mild neurocognitive disorder as a prodromal state of dementia is new. The clinical criteria developed by international experts, e.g., for frontotemporal dementia or Lewy body disease, are not yet sufficiently included in ICD-11. Biomarkers for the etiological diagnostics of dementia are also not mentioned, so that it is unclear which role they play in the disease classification in ICD-11. Due to the rapid development in the field of neurodegenerative diseases, regular updates would be desirable.

## Hintergrund

Bei Demenzerkrankungen findet aktuell ein konzeptueller Wandel statt. Bis vor Kurzem wurden sie in Bezug auf die Ätiologie syndromal diagnostiziert. Klinische Kriteriensätze wurden für alle häufigen Demenzformen (z. B. Lewy-Körper-Demenz, frontotemporale Demenzen) formuliert [7, 8]. In den letzten Jahren sind aber, insbesondere bei der Alzheimer-Krankheit, Biomarker für die Pathologie (Amyloid, Tau-Aggregation) hinzugekommen [6], wodurch eine gesicherte ätiologische Diagnose möglich ist. Bei anderen Demenzformen sind ebenfalls Biomarker in Entwicklung. Durch die Möglichkeit der biomarkerbasierten Erkennung der Alzheimer-Krankheit bereits im Stadium der leichten kognitiven Störung („mild cognitive impairment“, MCI) kann nach aktuellen diagnostischen Kriterien, die auch in die S3-Leitlinie Demenzen aufgenommen wurden, die Alzheimer-Krankheit bereits bei milden Symptomen, die noch nicht die Kriterien einer Demenz, aber eines MCI erfüllen, diagnostiziert werden [4, 9]. Die Entwicklung von einer klinisch-syndromalen hin zu einer biomarkerbasierten ätiologischen Diagnostik, inklusive Prodromalstadien, wird auch bei anderen Demenzerkrankungen stattfinden.

## ICD-10

Der syndromalen Einteilung folgend werden im Kapitel F0 der ICD-10 verschiedene Demenzformen genannt (Tab. 1). Die Demenz bei Alzheimer-Krankheit (F00.*) wird unterteilt in die mit frühem Beginn unter 65 Jahre (F00.0) und die mit spätem Beginn ab 65. Jahre (F00.1). Ferner kann die atypische oder gemischte Form (F00.2) und die Demenz bei Alzheimer-Krankheit, nicht näher bezeichnete (F00.9) codiert werden.

Bei der vaskulären Demenz (F01) werden die mit akutem Beginn (F01.0), die Multiinfarktdemenz (F01.1), die subkortikale vaskuläre Demenz (F01.2), die gemischt kortikal und subkortikale (F01.3), sonstige vaskuläre (F01.8) und nicht näher bezeichnet vaskuläre Demenz (F01.9) unterschieden.

Unter F02 werden Demenzen bei anderorts klassifizierten Erkrankungen gelistet. Hierzu gehören die Pick-Krankheit (F02.0), die Creutzfeldt-Jakob-Krankheit (F02.1), die Chorea Huntington (F02.2), das primäre Parkinson-Syndrom (F02.3), die HIV(„human immunodeficiency virus“)-Krankheit (F02.4) sowie weitere andernorts klassifizierte Erkrankungen (F02.8), z. B. die Lewy-Körper-Krankheit (s. unten).

Unter F03 werden nicht näher bezeichnete Demenzen und unter F04 das nichtalkoholisch und nicht durch andere psychotrope Substanzen ausgelöste organisch-amnestische Syndrom codiert.

**Tab. 1:** Codierung primärer Demenzerkrankungen und leichter kognitiver Störung in ICD-10 und ICD-11

ICD-10	ICD-11
**F00–F09: Organische, einschließlich symptomatischer psychischer Störungen**	**06: Neurokognitive Störungen**
*F00: Demenz bei Alzheimer-Krankheit*	*6D80 Demenz durch Alzheimer-Krankheit*
F00.0: mit frühem Beginn unter 65 Jahre	6D80.0: Demenz durch Alzheimer-Krankheit mit frühem Beginn
F00.1: mit spätem Beginn	6D80.1: Demenz durch Alzheimer-Krankheit mit spätem Beginn
F00.2: atypische oder gemischte Form	6D80.2: Alzheimer-Demenz, gemischter Typ, mit zerebrovaskulärer Krankheit 6D80.3: Alzheimer-Demenz, gemischter Typ, mit sonstiger nichtvaskulärer Ätiologie
F00.9: nicht näher bezeichnet	6D80.Z: Demenz durch Alzheimer-Krankheit, Beginn unbekannt oder nicht näher bezeichnet
*F01: Vaskuläre Demenz*	*6D81: Demenz durch zerebrovaskuläre Krankheit*
F01.0: Vaskuläre Demenz mit akutem Beginn	Spezifizierung von 8 Formen zerebrovaskulärer Krankheiten durch Postkoordination möglich
F01.1: Multiinfarktdemenz	–
F01.2: Subkortikale vaskuläre Demenz	–
F01.3: Gemischte kortikale und subkortikale vaskuläre Demenz	–
F01.8: Sonstige vaskuläre Demenz	–
F01.9: Vaskuläre Demenz, nicht näher bezeichnet	–
*F02: Demenz bei anderenorts klassifizierten Krankheiten*	*6D85: Demenz durch anderenorts klassifizierte Krankheiten (10 Krankheiten, Auswahl s. unten)*
F02.0: Demenz bei Pick-Krankheit	6D83: Frontotemporale Demenz (eigene Kategorie)
F02.1: Demenz bei Creutzfeldt-Jakob-Krankheit	6D85.5 Demenz durch Prionenerkrankung
F02.2: Demenz bei Chorea Huntington	6D85.1 Demenz durch Huntington-Krankheit
F02.3: Demenz bei primärem Parkinson-Syndrom	6D85.0 Demenz durch Parkinson-Krankheit
F02.4: Demenz bei HIV-Krankheit	6D85.3 Demenz durch HIV
F02.8: Demenz bei anderenorts klassifizierten Krankheitsbildern:	6D85.Y Demenz durch sonstige näher bezeichnete anderenorts klassifizierte Krankheiten
Lewy-Körper-Krankheit 14 weitere Krankheiten aus verschiedenen Bereichen	6D82: Demenz durch Lewy-Körper-Krankheit (eigene Kategorie)
*F03: Nicht näher bezeichnete Demenz*	*6D8Z: Demenz, nicht näher bezeichnete oder unbekannte Ursache*
*F04: Organisches amnestisches Syndrom, nicht durch Alkohol oder andere psychotrope Substanzen bedingt*	*6D72 Amnestische Störung*
*F06: Andere psychische Störungen aufgrund einer Schädigung oder Funktionsstörung des Gehirns oder einer körperlichen Krankheit*	–
F06.7: Leichte Kognitive Störung	6D71: Leichte neurokognitive Störung
*U63.-! Psychische und Verhaltensstörungen bei Demenz (7 Syndrome)*	*6D86 Verhaltensstörungen oder psychologische Störungen bei Demenz (7 Syndrome)*
**G30–G32: Sonstige degenerative Krankheiten des Nervensystems (Grunderkrankungen)**	**08: Störungen mit neurokognitiven Beeinträchtigungen als Hauptmerkmal**
*G30: Alzheimer-Krankheit*	*8A20: Demenz durch Alzheimer-Krankheit*
G30.0: Alzheimer-Krankheit mit frühem Beginn	–
G30.1: Alzheimer-Krankheit mit spätem Beginn	–
G30.8: Sonstige Alzheimer-Krankheit	–
G30.9: Alzheimer-Krankheit, nicht näher bezeichnet	–
*G31.0: Umschriebene Hirnatrophien (frontotemporale Demenz, Pick-Krankheit, progressive isolierte Aphasie)*	*8A21: Progressive fokale Atrophien* *8A23: Frontotemporale Lobärdegeneration*
*A81.0: Demenz bei Creutzfeldt-Jakob-Krankheit*	–
*G10: Demenz bei Chorea Huntington*	–
*G20.: Demenz bei primärem Parkinson-Syndrom*	–
*B22: Demenz bei HIV-Krankheit [Humane Immundefizienz-Viruskrankheit]*	–
*G31.82 Lewy-Körper-Krankheit*	*8A22: Lewy-Körper-Krankheit*

*HIV* „human immunodeficiency virus“, *ICD *International Statistical Classification of Diseases and Related Health Problems

F06 umfasst andere psychische Störungen aufgrund einer Schädigung oder Funktionsstörung des Gehirns oder einer körperlichen Krankheit. Hier kann die leichte kognitive Störung (F06.7) codiert werden, wenn eine zugrunde liegende Gehirnerkrankung oder körperliche Krankheit anderer Art benannt werden kann. MCI im Kontext der Alzheimer-Krankheit kann hier codiert werden, wobei die F06.7 nicht primär für Demenzerkrankungen gedacht ist.

Im neurologischen Kapitel findet sich die Alzheimer-Krankheit unter G30. Weitere codierbare Demenzformen sind die frontotemporale Demenz (G31.0), die Degeneration des Gehirns durch Alkohol (G31.2), die Chorea Huntington (G10), das primäre Parkinson-Syndrom (G20) und die Lewy-Körperchen-Krankheit (G31.82).

## ICD-11

In ICD-11 werden aktuelle diagnostische Klassifikationen neurodegenerativer Demenzerkrankungen aufgriffen (Tab. 1; [1]). In Kapitel 6 (Psychische Störungen, Verhaltensstörungen, neuromentale Entwicklungsstörungen) werden in dem Unterkapitel Neurokognitive Störungen die Demenzen aufgeführt. Aufgelistet werden hier die Demenz durch Alzheimer-Krankheit (6D80), eingeteilt in die mit frühem Beginn vor dem 65. Lebensjahr (6D80.0) und die mit spätem Beginn ab dem 65. Lebensjahr (6D80.1). Ferner werden Mischformen mit vaskulärer Pathologie (6D80.2) und mit anderen neurodegenerativen Pathologien (6D80.3) unterschieden. Durch die Postkoordinationsfunktion können den Mischformen spezifische Erkrankungen zugeordnet werden.

Einen eigenen Code hat die Demenz durch primär zerebrovaskuläre Krankheit (6D81). Hier können durch Postkoordination verschiedene Formen zerebrovaskulärer Erkrankungen mit jeweils eigenen Codes zugeordnet werden. Im Weiteren können Demenz durch Lewy-Körper-Krankheit (6D82), frontotemporale Demenz (6D83), Demenz durch psychoaktive Substanzen (6D84), inklusive Alkohol (6D84.0), Demenz durch Sedativa, Hypnotika oder Anxiolytika (6D84.1), durch flüchtige Inhalantien (6D84.2) und durch sonstige, nicht näher bezeichnete psychoaktive Substanzen (6D84.3) sowie Demenzen durch andernorts klassifizierte Krankheiten (6D85) codiert werden. Zu dem letzten Code sind neun Krankheiten gelistet, die jeweils über Postkoordination verknüpft werden können. Es bleiben noch die Demenz bei anderorts nicht klassifizierten Erkrankungen (D6586.Y) und die Demenz unbekannter Ursache (D6586.Z).

Alle Demenzen können über Postkoordination in leicht, mittelgradig und schwer eingestuft werden. Psychische und Verhaltenssymptome können einzeln codiert werden (psychotische Symptome 6D86.0, affektive Symptome 6D86.1, Angstsymptome 6D86.2, Apathie 6D86.3, Agitation oder Aggression 6D86.4, Enthemmung 6D86.5, Umherwandern 6D86.6, sonstige 6D86.Y, nicht näher bezeichnet 6D86.Z).

Neu ist die leichte neurokognitive Störung (6D71), die dem Konzept des MCI als frühe symptomatische Ausprägung einer neurodegenerativen Erkrankung vor dem Vollbild der Demenz entspricht. Über Postkoordination kann die leichte neurokognitive Störung mit spezifischen Krankheiten, z. B. der Alzheimer-Krankheit, verbunden werden. Von der leichten neurokognitiven Störung abzugrenzen ist die isolierte amnestische Störung (6D72).

Im neurologischen Kapitel 8 (Krankheiten des Nervensystems) sind Demenzen bei Störungen mit neurokognitiven Beeinträchtigungen als Hauptmerkmal aufgeführt. Hier können die Alzheimer-Krankheit (8A20), progressive fokale Atrophien (8A21), inklusive der posterioren kortikalen Atrophie (8A21.0), sonstige näher bezeichnete (8A21.Y) und nicht näher bezeichnete (8A21.Z) progressive fokale Atrophien, die Lewy-Körper-Krankheit (8A22), die frontotemporale Lobärdegeneration (8A23) und sonstige näher (8A2Y) oder nicht näher bezeichnete (8A2Z) Störungen codiert werden. Durch Postkoordination ist die Codierung einer Demenz mit Schweregrad für die Alzheimer-Krankheit, die Lewy-Körper-Krankheit und die frontotemporale Lobärdegeneration möglich. Die Kombinationsmöglichkeit dieser Krankheiten mit der leichten neurokognitiven Störung besteht nicht.

Im Kapitel 6 werden die codierbaren Entitäten mit Texten zur Symptomatik und zu Ursachen beschrieben, sodass ein narratives Verständnis für die Krankheiten und Syndrome entsteht. Im Kapitel 8 wird auf diese Beschreibungen verzichtet. In beiden Kapiteln werden keine konkreten diagnostischen Kriterien genannt und keine apparative oder Biomarkerdiagnostik erwähnt.

## Bewertung von ICD-11

In ICD-11 sind die Klassifikationen dem aktuellen Stand des Wissens in weiten Teilen angepasst und eine differenzierte Codierung von Demenzerkrankungen ist möglich [11]. Die häufigsten Formen von Demenzen, die auch in der S3-Leitlinie Demenz abgebildet sind, werden benannt [9].

## Aktualisierung der ätiologischen Kategorien und Postkoordination

Unter Beibehaltung der syndromalen Einteilung der Demenzformen wurden Begriffe im Vergleich zu ICD-10 aktualisiert, differenzierte Codiermöglichkeiten gemischter Demenzformen geschaffen und nicht mehr aktuelle Begriffe (z. B. Pick-Krankheit) verlassen.

Eine wesentliche Weiterentwicklung ist die kapitelübergreifende Postkoordination, durch die auf Codes anderer Kapitel zurückgegriffen werden kann, um ein Krankheitsbild möglichst genau zu erfassen. Dies wird z. B. bei der Demenz durch zerebrovaskuläre Krankheit (6D81) deutlich, bei der durch Postkoordination mit dem neurologische Kapitel 8 eine genaue ätiologische Beschreibung der zugrunde liegenden zerebrovaskulären Krankheit möglich ist.

Allerdings bleibt auch ICD-11 in weiten Teilen auf der Ebene der Syndrome [11]. Dies ist bei der Alzheimer-Krankheit zu kritisieren, da ätiologische Biomarker auf Basis von Liquor und Positronenemissionstomographie (PET) ausgereift und im klinischen Einsatz sind. Da die syndromale Diagnostik eine erhebliche Unschärfe bezüglich der zugrunde liegenden Pathologie hat, ist zu fordern, dass die Ätiologie über Biomarkerdiagnostik definiert wird. Durch die Entwicklung blutbasierter Biomarker wird sich die Zugänglichkeit dieser Diagnostik weiter verbessern [10]. Hinzu kommt, dass bei der Alzheimer-Krankheit erste Therapien verfügbar sind, die eine Bestimmung der Amyloidpathologie mittels Biomarkern voraussetzen [5]. Gleiche Entwicklungen finden auch bei anderen neurodegenerativen Erkrankungen statt, sind allerdings noch nicht so weit fortgeschritten.

Unabhängig davon ist zu kritisieren, dass sich die klinisch-syndromale Zuordnung nicht eng an den von internationalen Experten entwickelten klinischen Kriterien für die einzelnen neurodegenerativen Erkrankungen, z. B. der frontotemporalen Demenz inklusive Unterformen oder der Lewy-Körper-Krankheit, orientiert [7, 8]. Kapitel 6 greift diese Kriterien zwar allgemein, aber nicht exakt auf. Hierdurch wird die Spezifität der klinisch-syndromalen Diagnostik, die durch die Expertenkriterien erreicht werden könnte, nicht ausgeschöpft und verhindert, dass die ICD-11-Codierung in klinischen Studien mit neuen Therapieansätzen eingesetzt werden kann.

Es gibt auch nicht adäquate Einteilungen, die fortgetragen wurden. Die Aufteilung der Alzheimer-Krankheit in eine früh und eine spät beginnende Form, die hilfsweise zwischen seltenen monogenen und sporadischen Varianten unterscheiden soll, lässt sich so nicht aufrechterhalten. Es gibt sowohl monogene Fälle der Alzheimer-Krankheit mit einem Beginn nach dem 65. Lebensjahr als auch nichtmonogene Fälle mit einem Beginn davor [12]. In der Diagnostik und Behandlung gibt es keinen grundsätzlichen Unterschied zwischen den Altersgruppen. Ätiologisch wäre eher eine altersunabhängige Einteilung in monogene und nichtmonogene Fälle sinnvoll. Möchte man die Altersunterteilung für statistische Fragen verwenden, müsste sie für alle Demenzformen angewendet werden.

Ein weiterer Aspekt ist das Fehlen der syndromalen Beschreibung atypischer Formen der Alzheimer-Krankheit. Die logopenische Aphasie, die meistens auf die Alzheimer-Krankheit zurückzuführen ist, wird unter den frontotemporalen Demenzen codiert [2]. Die posteriore kortikale Atrophie, die auch überwiegend durch Alzheimer-Krankheit verursacht wird, kann im Kapitel 8 codiert werden, allerdings ohne Bezug zur Alzheimer-Krankheit [3]. Eine Verbindung durch Postkoordination ist in beiden Fällen nicht möglich. Unter der Annahme, dass Betroffene mit diesen atypischen Ausprägungen der Alzheimer-Krankheit von Antiamyloidtherapien profitieren könnten, ist diese Klassifikation problematisch.

## Versorgungsrelevante Codierungen

Eine überzeugende Neuerung in ICD-11 ist die Codierung des Schweregrads der Demenz in leicht, mittelgradig und schwer, welche für Versorgungsplanung und -kosten von großer Bedeutung ist. Ebenfalls gut ist, dass einzelne psychische und Verhaltenssymptome durch Postkoordination für jede Demenzform codiert werden können. Dies ist für die Versorgungsplanung relevant, da psychische und Verhaltenssymptome einen großen Teil des medizinischen und pflegerischen Aufwands bedingen. In ICD-10 ist die Codierung dieser Symptome nur in dem Kapitel U möglich und wird kaum angewendet.

## Leichte neurokognitive Störung

Ein zentraler Fortschritt von ICD-11 ist die Einführung der leichten neurokognitiven Störung – die erstmal bereits in der 5. Version des Diagnostic and Statistical Manual of Mental Disorders (DSM-5) eingeführt wurde – mit der Möglichkeit über Postkoordination diesem Syndrom eine spezifische Ätiologie zuzuordnen. Hierdurch wird es möglich, die Diagnose der Alzheimer-Krankheit zu einem Zeitpunkt zu stellen, an dem eine betroffene Person z. B. von einer krankheitsmodifizierenden Therapie profitieren kann. Mit dieser Diagnose schließt die Versorgung zur Forschung auf, die seit langer Zeit auf die Prädemenzphase neurodegenerativer Erkrankungen in der Diagnostik- und Therapieentwicklung fokussiert [5].

## Vergleich der Kapitel 6 und 8

Das psychiatrische Kapitel 6 verwendet weiterhin Syndrome als Leitkriterien (z. B. frontotemporale Demenz), wohingegen das neurologische Kapitel 8 Krankheitsbegriffe (z. B. Alzheimer-Krankheit) oder morphologische Klassifikationen (z. B. frontotemporale Lobärdegeneration) ohne Bezug zur syndromalen Ausprägung verwendet. Durch die syndromale Herangehensweise in Kapitel 6 besteht ein direkterer Bezug zum Krankheitsschweregrad und damit Versorgungsaufwand, gleichzeitig wird aber die ätiologische Unschärfe fortgetragen. Durch die Postkoordination kann diese zum Teil überwunden werden. Kapitel 8 entspricht der ätiologischen Ausrichtung der Diagnose, lässt aber keine Rückschlüsse auf stadienspezifische Häufigkeiten oder Behandlungsindikationen zu. Allerdings besteht hier die Möglichkeit, einzelne Diagnosen über Postkoordination mit dem Code für eine leichte, mittelschwere oder schwere Demenz zu verbinden.

## Fazit für die Praxis

- Die International Statistical Classification of Diseases and Related Health Problems 11 (ICD-11) stellt einen klaren Fortschritt gegenüber ICD-10 dar, v. a. durch die verbesserten Möglichkeiten die Syndrome mit Ätiologien zu verbinden, die versorgungsrelevanten Krankheitsausprägungen über Schweregrade und psychische und Verhaltenssymptome zentraler darzustellen und die Möglichkeit die leichte neurokognitive Störung im Kontext neurodegenerativer Erkrankungen zu diagnostizieren.
- Bei weiterhin bestehender klinisch-syndromaler Diagnostik ist eine Limitation, dass sich ICD-11 nur grob an wissenschaftlich begründete klinische Kriterien einzelner Krankheitsbilder hält und stattdessen allgemeinere Beschreibungen aufführt, die mit größerer diagnostischer Ungenauigkeit einhergehen, und daher unzureichend als Einschlusskriterien für klinische Prüfungen mit neuen Therapieansätzen sind.
- Eine biomarkerbasierte ätiologische Diagnostik von Demenzerkrankungen sollte in die Weiterentwicklungen von ICD-11 integriert werden.
